# Clear Theories Are Needed to Interpret Differences: Perspectives on the Bilingual Advantage Debate

**DOI:** 10.1162/nol_a_00038

**Published:** 2021-11-11

**Authors:** Angela de Bruin, Anthony Steven Dick, Manuel Carreiras

**Affiliations:** Department of Psychology, University of York, York, United Kingdom; Basque Center on Cognition, Brain and Language (BCBL), Donostia-San Sebastián, Spain; Department of Psychology, Florida International University, Miami, FL, United States; University of the Basque Country, Bilbao, Spain; Ikerbasque, Basque Foundation for Science, Bilbao, Spain

**Keywords:** bilingual advantage, executive control, bilingualism, inhibition, language control, brain plasticity

## Abstract

The heated debate regarding bilingual cognitive advantages remains ongoing. While there are many studies supporting positive cognitive effects of bilingualism, recent meta-analyses have concluded that there is no consistent evidence for a *bilingual advantage*. In this article we focus on several theoretical concerns. First, we discuss changes in theoretical frameworks, which have led to the development of insufficiently clear theories and hypotheses that are difficult to falsify. Next, we discuss the development of looking at bilingual experiences and the need to better understand *language* control. Last, we argue that the move from behavioural studies to a focus on brain plasticity is not going to solve the debate on cognitive effects, especially not when brain changes are interpreted in the absence of behavioural differences. Clearer theories on both behavioural and neural effects of bilingualism are needed. However, to achieve this, a solid understanding of both bilingualism and executive functions is needed first.

## INTRODUCTION

Speaking more than one language is advantageous in this multilingual and highly interconnected world. Multilinguals can communicate directly with speakers of other languages and have access to other cultures, among other benefits. Apart from these communication advantages, bilingualism has been claimed to improve other cognitive domains, in particular executive functioning (e.g., Bialystok et al., 2004). The underlying idea is that executive functions would be used and developed more strongly in bilinguals than in monolinguals. Bilinguals are said to acquire better inhibitory control and monitoring skills than monolinguals because they need to inhibit the irrelevant language, monitor the surroundings, and resolve conflicting information (e.g., Bialystok et al., 2004). In addition, bilinguals who frequently switch between languages in daily life may have better task-switching skills (e.g., Prior & Gollan, 2011). Furthermore, cognitive benefits of bilingualism have been linked to delays in the onset of diseases such as dementia and to less decline associated with healthy aging, with possible implications for public health (Bialystok et al., 2016; Perani et al., 2017). This attractive idea termed *bilingual advantage* (e.g., Kroll & Bialystok, 2013) has been quickly adopted by the mass media publishing headlines and statements such as: “Bilingual children and adults experience significant health benefits” (Huffington Post, 2013); “Bilingual students advance faster in middle school” (Rahhal, 2018).

Despite the popularity of this topic in the media and the emphasis on societal benefits, the evidence for enhanced cognitive functioning in bilinguals is also widely questioned. In this article, we will start with a brief overview of the evidence for a bilingual advantage. (Note that we will be using the term *bilingual advantage* to refer to *cognitive* advantages.) We will focus on bilingualism and cognition in healthy participants. This has been the focus of many recent systematic reviews and meta-analyses (e.g., Antoniou, 2019; Bialystok, 2017; Donnelly et al., 2019; Hilchey et al., 2015; Lehtonen et al., 2018; Paap, 2019). Critically, we will focus on several theoretical issues that are important to consider to move the debate forward. First, we discuss the continuous changes in theoretical frameworks and issues when testing these frameworks. Next, we discuss how differences in executive functioning have been associated with individual differences between bilinguals. Although this is a promising way forward, we argue that this can only be examined with a solid understanding of individual differences in *language* control. Third, recent years have seen an increase in neuroimaging studies comparing bilinguals and monolinguals on executive control tasks. We argue that, while these neuro-imaging studies are interesting in their own respect, they cannot solve the bilingual advantage debate without behavioural evidence and without clear predictions about the specific brain regions and brain circuits that should reveal bilingual-monolingual differences in executive control. For this field to progress, more specific theories and hypotheses need to be formed regarding the behavioural and neural relationship between bilingualism and executive control. We will posit that two developments are needed to achieve this. First, to develop theories, a better understanding of bilingualism and bilingual *language* control is needed. Second, multi-lab studies with clear preregistered hypotheses are needed to reliably interpret the data across different types of bilinguals.

## BEHAVIOURAL COMPARISONS BETWEEN BILINGUALS AND MONOLINGUALS

Several early studies comparing bilinguals and monolinguals showed enhanced performance in bilinguals on various tasks assumed to measure executive functioning (e.g., Bialystok et al., 2004; Bialystok et al., 2008; Bialystok & Martin, 2004; Costa et al., 2009; Costa et al., 2008). In contrast, there are also many studies reporting no differences between bilinguals and monolinguals on executive control tasks (e.g., Antón et al., 2014; Antón et al., 2016; Duñabeitia et al., 2014; Gathercole et al., 2014; Paap & Greenberg, 2013), especially in studies using larger sample sizes (e.g., Antón et al., 2019; Dick, Garcia et al., 2019; Duñabeitia et al., 2014; Nichols et al., 2020; Paap & Greenberg, 2013).

A more comprehensive overview of the literature on this topic has been provided in recent systematic reviews (e.g., Antoniou, 2019; Bialystok, 2017; van den Noort et al., 2019) and meta-analyses (e.g., Donnelly et al., 2019; Grundy, 2020; Gunnerud et al., 2020; Lehtonen et al., 2018; Paap, 2019). While there is both evidence for and against a bilingual cognitive advantage, recent meta-analyses taking into account publication bias (e.g., Lehtonen et al., 2018) conclude that there is no strong or consistent evidence for enhanced executive functioning in bilinguals. Similar conclusions were reached in a meta-analysis on children (Gunnerud et al., 2020), although a small but significant effect on switching was found. These reviews and meta-analyses also attempted to go beyond a “yes/no” answer, acknowledging that studies differ in, amongst others, the type of bilinguals tested and the type of executive control tasks used. These are all variables that can potentially modulate performance of both bilinguals and monolinguals on executive control tasks. Currently, however, there is no consistent evidence *across studies* for a modulating role of, for example, specific bilingual experiences or the type of task used (Lehtonen et al., 2018).

In addition, it is frequently claimed that differences may not be captured in young adults because they perform at ceiling, but that cognitive development and decline should allow for effects of bilingualism to occur in children and older adults (e.g., Kroll & Bialystok, 2013; Grundy, 2020). Meta-analyses, however, have found comparable (null) results in different age groups (e.g., younger and older adults, Lehtonen et al., 2018; children and young adults, Donnelly et al., 2019), suggesting that evidence for a bilingual advantage is limited not only in young adults, but also in children and (healthy) older adults. Interestingly, it has also been proposed that the effects of bilingualism on attentional processes could be observed already during infants’ first year of life (Comishen et al., 2019; Kovács & Mehler, 2009). However, data from a recent Registered Report (Kalashnikova et al., 2021), show that bilingual and monolingual infants’ performance did not differ in attentional control.

In sum, systematic reviews and meta-analyses thus suggest that a cognitive bilingual advantage is at best small and may only exist in very specific circumstances or for specific types of bilinguals. We will discuss three recent developments that have been proposed as future avenues for research on bilingualism and executive control. First, we will consider changes in theoretical frameworks. Second, we will discuss the role of individual bilingual experiences. Third, we will review recent neuroimaging research used to examine the potential relationship between bilingualism and executive control.

## THEORETICAL FRAMEWORK AND DATA INTERPRETATION

Much past and recent work on bilingual-monolingual differences focuses on inhibition costs. Studies looking at inhibitory control typically include conditions with incongruent trials (presenting information that interferes with the expected response) and congruent trials (presenting information that is compatible with the expected response). Participants usually need more time to respond to incongruent than congruent conditions, a difference that is taken to reflect an inhibition cost. Some studies observe bilingual-monolingual differences on these inhibition costs (e.g., Pelham & Abrams, 2014) while others do not (e.g., Paap & Greenberg, 2013). Others find that within the study, effects may be task dependent (e.g., Woumans et al., 2015, showed a larger monolingual *inhibition cost* on one task but longer monolingual *overall* reaction times [RTs] on another task). The initial framework regarding bilingualism and executive control focused on inhibition. According to the initial hypothesis focusing on inhibition, a bilingual constantly needs to inhibit one of their languages in daily life, which should lead to non-verbal inhibitory control advantages (e.g., Bialystok et al., 2004). Theories focusing on inhibition would posit that bilingual-monolingual differences should occur on measures of inhibition costs, such as the difference between incongruent and congruent trials described above. This offers a testable hypothesis.

One major challenge when looking at inhibition costs, however, is that tasks reporting these costs often show low correlations (cf. Paap & Greenberg, 2013; Rouder et al., 2019). The idea that inhibition is a unitary construct has therefore been criticised (Rey-Mermet et al., 2018), raising the point that tasks might not measure the underlying construct of inhibition but rather the “highly task-specific ability to resolve the interference arising in that task” (Rey-Mermet et al., 2018, p. 515). Following this account, it could be argued that the mixed results in the literature on bilingualism might reflect task-dependent effects, with bilinguals having an advantage on a certain type of task-specific interference resolution. If this is true, certain tasks should stand out (e.g., a Simon task might be more likely to reveal a bilingual-monolingual difference than, e.g., a flanker task). However, current comparisons across studies (e.g., Lehtonen et al., 2018) do not show consistent evidence for such differences between *inhibition tasks*. Furthermore, based on our current understanding of these different tasks, it is unclear exactly which types of task-specific interference resolution would be most likely to be affected by bilingualism (cf., e.g., Blumenfeld & Marian, 2014, and Paap et al., 2019). To be able to formulate any theories or hypotheses that take into consideration task-specific types of inhibition/interference resolution, we need a much better understanding of what these different tasks actually measure.

A second explanation of low correlations between inhibition tasks is that they measure the same underlying construct but that task outcomes are influenced by task-specific features that add noise. This issue could, at least to some extent, be overcome through analyses using latent variables (cf. Friedman, 2016; Rouder et al., 2019). If there is an underlying construct, this allows researchers to examine whether there is a bilingual-monolingual difference on *that construct* as measured through multiple tasks rather than on one specific task that might be influenced by task-specific features. Similar approaches can be used for other constructs of interest that have been tested in relation to bilingual-monolingual differences (e.g., task switching, Prior & Gollan, 2011; working memory, e.g., Engel de Abreu, 2011).

While the initial research (and much of the recent research) focuses on specific aspects of executive function, such as inhibition or shifting, the hypotheses regarding bilingualism and executive control are undergoing constant changes. This is driven by studies finding group differences on overall RTs in conflict tasks (e.g., Emmorey et al., 2008), without a difference in inhibition costs (but cf. Bialystok et al., 2004). New theories were therefore proposed that focused on an advantage in conflict monitoring rather than inhibition, possibly related to a bilingual's need to monitor the circumstances to select the current target language (e.g., Costa et al., 2009). These theories can offer testable hypotheses if they include a baseline condition. For instance, accounts on conflict monitoring posit that a bilingual advantage should only occur in conditions involving both conflict and non-conflict trials (i.e., requiring more conflict monitoring; cf. Costa et al., 2009). Such advantage would not be expected in conditions that do not require conflict monitoring. However, studies finding overall RT differences between bilinguals and monolinguals on conflict tasks do not always include a baseline condition. Without such a baseline condition, it is difficult to interpret whether RT differences are really due to conflict monitoring. For example, it might be that one group is faster *in general*. Adding a baseline task without conflict could show whether bilingual-monolingual RT differences are specific to conflict monitoring (RT differences in the conflict but not in the baseline task) or related to differences in general processing speed (RT differences in all tasks, even those without conflict). We therefore recommend the inclusion of simple baseline conditions without conflict. For example, a flanker task could include a baseline condition in which participants simply respond to one arrow presented in the centre of the screen. RTs (and potential group differences) in this baseline task can then be compared to the flanker task requiring conflict monitoring through presentation of congruent and incongruent (conflict) trials.

In recent years, a more holistic approach has been advocated (e.g., Kroll & Bialystok, 2013) that focuses on executive functions as a whole rather than specific subcomponents such as inhibition or switching. Different terms have been used, including enhanced *cognitive flexibility* (Kroll & Bialystok, 2013) and *executive attention* (Bialystok, 2017). This executive attention system is described as a continuous, central, domain-general system in which memory and attention interact to allow for complex cognition (Bialystok, 2017). The danger with some newer frameworks, however, is that they become unprofitably vague. That is, executive attention is seen as a continuous mechanism that is involved in all sorts of complex cognition. Differences in executive attention can occur on a wide range of tasks and measures without a clear theory or hypotheses as to *when* and *where* these effects should be observed (cf. Hartsuiker, 2015; Laine & Lehtonen, 2018).

This poses problems for studies that test bilinguals and monolinguals on different tasks, that look at conflict costs and overall RTs, and that measure RTs and accuracy, and that then find a bilingual-monolingual difference on one measure only. This type of research should either be hypothesis-driven or should avoid drawing conclusions about individual tasks. When there is a clear theory and hypothesis, task-specific patterns can be interpreted. For example, a researcher might be interested in assessing the role of verbal versus nonverbal stimuli based on the hypothesis that bilinguals experience language interference in tasks using verbal materials. A bilingual disadvantage on the verbal but not on the nonverbal task could then be explained following their hypothesis. When such theory or hypothesis is not present (e.g., when a Simon and flanker task are included, without a clear hypothesis about potential task differences), the danger is that *any* difference on *any* task would be interpreted as evidence for a bilingual advantage, with a focus on the study’s positive findings. Without a clear theory or hypothesis about what different tasks/measures indicate, how they differ or compare, and how they might show different patterns in bilinguals and monolinguals, we should be careful that we do not focus too much on or overinterpret the measure that shows a positive finding. In the absence of clear hypotheses about task-specific effects, latent-variable analyses (cf. Friedman, 2016; Rouder et al., 2019) might be preferable. This avoids having to generate post hoc explanations for task differences that might be the result of noise added by task-specific features.

## INDIVIDUAL DIFFERENCES BETWEEN BILINGUALS

In addition to tasks potentially influencing bilingual-monolingual differences, the type of bilinguals tested might play an important role. This potential influence of individual differences in bilingualism has been the focus of recent research. One advantage of this approach is the move away from presenting bilinguals and monolinguals as homogenous groups that are the same across populations and studies. Comparing a group of bilinguals to a group of monolinguals inevitably requires the researchers to define where one group ends and the other starts. While the definition of a monolingual might seem straightforward (e.g., a person who can only speak one language), this is often not as easy as it seems. “Monolinguals” sometimes include participants who have learned and/or have some proficiency in another language (e.g., Paap & Greenberg, 2013). Furthermore, dialect users are often classified as monolinguals (cf. Kirk et al., 2018) and monolinguals living in a linguistically diverse environment might differ from those living in more monolingual environments (cf. Bice & Kroll, 2019). Therefore, it might not (always) be possible to make a clear categorical distinction between bilinguals and monolinguals. Furthermore, a comparison between one group of bilinguals and one group of monolinguals creates the suggestion that there are no individual differences within those groups. Within bilinguals there are many individual differences (e.g., proficiency, age of acquisition, use, switching) and one’s personal language experiences have been argued to influence language control and consequently executive functions. These individual differences can and need to be studied in relation to executive functions. This can be done by comparing well-defined groups of bilinguals (e.g., a group of bilinguals with a high proficiency level in both languages versus a group with a lower proficiency in their second language) and/or by treating bilingualism and bilingual experiences (e.g., proficiency) as a continuum. Several language experiences have been studied in relation to executive function, including age of acquisition (e.g., Luk, De Sa, & Bialystok, 2011); proficiency (e.g., Singh & Mishra, 2013); and modality (e.g., Emmorey et al., 2008). Recent frameworks focus on language use and switching (cf. Blanco-Elorrieta & Pylkkänen, 2018). Green and Abutalebi’s (2013) Adaptive Control Hypothesis focuses on how a bilingual’s language use can shape both language control and executive functions, depending on the language environment they find themselves in. For example, a bilingual who spends much time in more controlled dual-language environments that require them to switch languages in response to cues (e.g., interlocutors) might have more need for and develop interference suppression and goal maintenance more strongly than a bilingual who can freely switch with other bilinguals who speak the same languages. The research comparing different types of language switchers has shown mixed effects (e.g., Paap et al., 2017; Prior & Gollan, 2011; Verreyt et al., 2016). To some extent mixed results might be due to the way language switching is measured and the type of switchers that are compared. For example, looking at frequency of switching might not consider that bilinguals who switch frequently might do so very differently. Thus, in addition to considering switching frequently, type of switching (e.g., in response to external cues versus free dense code switching) needs to be considered (Green & Abutalebi, 2013).

Crucially, though, the hypothesis that certain types of bilinguals might be more likely to show cognitive advantages than others is based on language control depending on bilingual experiences. The Adaptive Control Hypothesis (Green & Abutalebi, 2013) describes how these experiences might moderate both language control and executive control, but there is very little empirical work to assess influences on language control. Influences on executive control *depend* on understanding language control. To understand *transfer of training* (from language to executive control), we first need to understand the *training* itself (i.e., language control). To facilitate this, we need more detailed descriptions and measures of bilingual participants to examine the influence of bilingual experiences within and across studies (cf. de Bruin, 2019; Surrain & Luk, 2019). Recent work has started to suggest that language control might be shaped by the interactional context (e.g., Blanco-Elorrieta & Pylkkänen, 2017; de Bruin et al., 2018). There is also some emerging work comparing different types of bilinguals on, for example, the processing of code switches (e.g., Beatty-Martínez & Dussias, 2017). However, far more research is needed to understand how language experiences can shape language control before we can form more exact theories about which language experiences might shape (components of) executive control.

## BRAIN PLASTICITY

In addition to behavioural studies, there is now an increasing number of studies focusing on brain differences between bilinguals and monolinguals (cf. Bialystok, 2017; Grundy et al., 2017; Vīnerte & Sabourin, 2019). Other studies have shown that similar brain regions might be involved in language control and inhibitory control (cf. Abutalebi & Green, 2007) or in language and task switching (De Baene et al., 2015), which has been taken to suggest a close relationship between language and executive control. However, while many brain differences have been observed between bilinguals and monolinguals, there is currently no consistent evidence that specific control-related regions show bilingual-monolingual differences *across studies* (see García-Pentón et al., 2016). We do not aim to provide an exhaustive review of the neuroimaging literature here. What we do review is designed to show that the same issues described above for behavioural research also apply to neuroimaging studies. In addition, we will highlight additional challenges that need to be addressed to be able to interpret neuroimaging studies. We will first analyse recent work assessing EEG, followed by structural MRI and functional MRI (fMRI) research on bilingualism and executive functions.

### Electrophysiological Evidence

EEG (Electroencephalography) provides a rich and complex brain measure to investigate the potential influence of bilingualism on other cognitive processes such as executive control. The event related potentials (ERPs) that result from time locking the EEG signal to a particular event offer latency, amplitude, polarity, and topography as potential variables. Interestingly, some of these variables, like latency, are appropriate to capture early cognitive effects and the time course of different processes given its high temporal resolution. Nonetheless, differences between monolinguals and bilinguals in ERP amplitudes in the several different components (e.g., N200, P300, N400, ERN, etc.) allow different interpretations depending on whether an amplitude increase or decrease is associated with more efficient cognitive processes. At this point, ERP evidence for or against cognitive processing benefits for bilinguals versus monolinguals is limited and mixed (see Cespón & Carreiras, 2020).

As an overall strategy, Cespón and Carreiras (2020) reviewed the effects of tasks used to measure executive functions (e.g., Simon, flanker, Stroop tasks) on the latency and amplitude of different ERP components (N200, P300, N400/N450, ERN). This allowed them to establish hypotheses about whether an increase or decrease in amplitude was related to more or less efficient cognitive processes independently of bilingualism. Taking this knowledge into account they hypothesized how bilingualism is expected to modulate effects (e.g., latency, amplitude) of a particular component if a bilingual advantage were present. Based on this review per ERP component, they offer two main recommendations for the way differences in latency and amplitude (e.g., larger or smaller amplitude) should be interpreted when comparing bilinguals and monolinguals on these tasks. First, only specific differences in ERP latency and/or amplitude can be used to support claims regarding enhanced executive-control efficiency in bilinguals. Cespón and Carreiras (2020) reviewed literature on executive control assessing different ERP components and formulated hypotheses for bilingual-monolingual differences and interpretations depending on the direction of a difference (i.e., how bilingualism will modulate the established latency/amplitude effects and the corresponding interpretation). These hypotheses can guide new ERP research on bilingualism and executive control, allowing researchers to establish a priori hypotheses based on previous work linking specific changes in ERPs to more or less efficient cognitive processes.

Second, in their review Cespón and Carreiras (2020) recommend that researchers carry out correlational analyses of ERP amplitude with behavioural performance. This is important to establish whether potential ERP differences form a direct reflection of bilingual advantages/disadvantages. When there is a correlation between the behavioural difference and the ERP difference (e.g., a smaller behavioural cost in combination with a decreased amplitude for bilinguals), this supports the interpretation that there is an advantage for bilinguals on this task. When such correlation is not observed (or when there is an ERP difference without a behavioural difference), it suggests that bilinguals and monolinguals might differ in the way they process information but without a directly associated bilingual *advantage*.

### Magnetic Resonance Imaging Evidence

Functional magnetic resonance imaging (fMRI) and structural MRI (including diffusion-weighted imaging (DWI) measures and morphologic measures recovered from T1- and T2-weighted MRI scans), as well as related functional imaging methods such as functional near-infrared spectroscopy (fNIRS) and magnetoencephalography (MEG), have been extremely useful for understanding brain plasticity and development, and have been informative for mapping the functional specialization of different brain regions. Several of these methods have also been used to examine brain differences in structure or function that result from bilingual experience as it relates to differences in brain networks associated with executive function (see Bialystok et al., 2012; Costa & Sebastián-Gallés, 2014; García-Pentón et al., 2016; Grundy et al., 2017; Pliatsikas & Luk, 2016 for reviews). Rather than giving a review of the fMRI literature, we will discuss the key issues affecting fMRI research on bilingualism and executive function.

#### Theoretical framework

Most of the MRI studies on bilingual-monolingual differences or differences between bilinguals in executive function have framed their investigations within the context of cognitive and neural models such as the Inhibitory Control model (Green, 1998), and more recent modifications formulated in the Adaptive Control Hypothesis (Green & Abutalebi, 2013), or the bilingual anterior-to-posterior and subcortical shift (BAPSS) model (Grundy et al., 2017). Theoretical models are critically important for prediction and interpretation of activation differences across groups of bilinguals and monolinguals. This is because neurobiological models of language and executive function in monolingual individuals are themselves complicated, and there is debate within each subfield about which grey matter regions and white matter tracts are critical to networks underpinning each process. In the field of language neurobiology, for example, neural networks for speech production and speech perception are only partially overlapping, and the issue gets more complicated at higher levels of language processing. Thus, at the sentential and discourse levels, much more of the brain, on both hemispheres, is recruited, especially in situations where syntactic and semantic constructions are more complex, and in cases where pragmatics come into play (Hagoort, 2019). When these latter linguistic processes are brought to bear, it is reasonable to argue that higher-level control processes might be recruited to navigate the additional semantic, syntactic, and pragmatic challenges of communicating in more than one language. Indeed, such demands may not be universal, but may emerge only in specific contexts or situations—that is, any advantages emerge not from being bilingual, but from the ways in which languages need to be controlled in situational contexts (e.g., Blanco-Elorrieta & Pylkkänen, 2018). The question at the neurobiological level, though, is what this might look like in terms of changes in regional activity and network dynamics. Thus, some sort of framing model is needed to interpret any potential differences.

**Figure 1.**
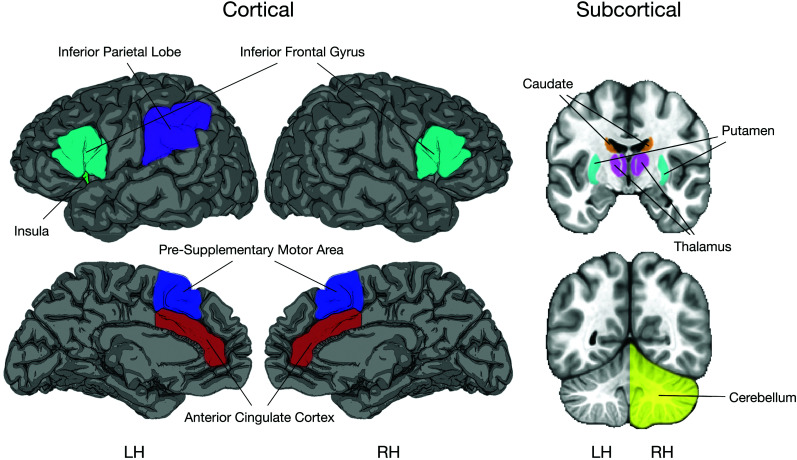
Brain regions comprising the Adaptive Control Model, based on Green and Abutalebi (2013). Cortical and subcortical brain regions are proposed to be involved in language control and language switching, and in implementing various control processes proposed by the Adaptive Control Model. LH = Left Hemisphere; RH = Right Hemisphere.

The Adaptive Control hypothesis is designed to address these issues, as its central goal is to “identify a set of language control processes that support conversation in different interactional contexts, articulate the relative demands of these contexts on these processes, and spell out the neural bases of adaptive changes” (Green & Abutalebi, 2013, p. 516). In different interactional contexts, potentially competing linguistic representations generated across languages may emerge and require resolution at multiple levels and timepoints in the process of producing and understanding language. Control processes such as goal maintenance and interference control (i.e., conflict monitoring and interference suppression) are likely to be brought to bear in such situations, and in some situations other processes such as selective response inhibition and task engagement and disengagement might be required. A network of brain regions is proposed to implement these component processes, mainly during language production (Green & Abutalebi, 2013). These include bilateral inferior frontal gyrus, inferior parietal, insula, dorsal striatum, thalamus, right cerebellum, anterior cingulate cortex, and pre-supplementary motor area (pre-SMA; see Figure 1). The expectation, from the perspective of the Adaptive Control model, is that *adaptive effects should be expressed in these regions that mediate control demands in bilingual contexts where these specific demands are high*.

A complementary neural model that has also been proposed is the BAPSS (Grundy et al., 2017). The central tenet of this model is that, for nonverbal executive tasks, “bilingualism is associated with a model of efficient brain recruitment” in the form of less recruitment of “frontal and executive regions” and greater recruitment of “posterior/subcortical regions” (p. 190). However, other than naming the dorsolateral prefrontal cortices and anterior cingulate cortices, the model is rather vague on what are the “basal ganglia and posterior regions.” In contrast to the Adaptive Control Model, which does specify a number of specific regional expectations, the BAPSS model is vague to the point where a number of findings can still fit within the model. As such, it does provide a framework for investigation, but further refinement of the specific regions proposed to be affected by bilingual experience would improve its usefulness.

Several fMRI studies have observed monolingual-bilingual differences in the regions described in these models (with some of these studies published before and forming the basis of these models). Studies focusing on cortical thickness or regional volume have reported differences between monolinguals and bilinguals at the whole-brain level (Grogan et al., 2012; Klein et al., 2014; Mechelli et al., 2004; Pliatsikas et al., 2014; Ressel et al., 2012; Zou et al., 2012), and some report the difference in brain regions associated with language control/executive control in, for example, the Adaptive Control Hypothesis (e.g., left inferior parietal lobule in Mechelli et al., 2004; also see Abutalebi et al., 2015; left caudate nucleus in Klein et al., 2014; Zou et al., 2012). Investigations based on region of interest more reliably find differences in such regions (Abutalebi et al., 2015) and in others (e.g., auditory cortex; Ressel et al., 2012), although several studies also report no reliable differences between monolingual and bilingual groups when the groups are examined at the whole-brain level (Gold et al., 2013; Grogan et al., 2012; Ressel et al., 2012).

Similar studies examining differences in white matter diffusion properties, whether in white matter regions or in specifically defined fibre pathways, have also been reported. Such findings are, in some ways, even more difficult to interpret than regional morphometric differences with respect to how they relate to bilingual executive function advantages. This is because the nature of structural connections in white matter is difficult to ascribe to singular functions. First, although there is an emerging consensus regarding which fibre pathways comprise a “language connectome” (Dick et al., 2014), there is not a one-to-one mapping between function and pathway, and some pathways are associated with both language and executive function (Dick, Garic et al., 2019). Second, the white matter of the cortex is overwhelmingly populated with *crossing-fibres* from multiple dissociable projections. Thus, regional differences in white matter may indicate structural differences in multiple crossing fibre pathways, or at least it may be difficult to ascertain which pathway is contributing to the differences. Interpretation of group differences in white matter are therefore a cautious exercise.

Considered alongside these important caveats, there are several findings of note in the literature looking at structural differences (see García-Pentón et al., 2016, for review). It is encouraging to note that reliable differences are found in white matter and fibre pathways associated with processing language in monolingual populations (e.g., inferior fronto-occipital fasciculus (IFOF), inferior longitudinal fasciculus (ILF), uncinate fasciculus (UF), and superior longitudinal fasciculus (SLF); Cummine & Boliek, 2013; Gold et al., 2013; Luk, Bialystok et al., 2011; Mohades et al., 2012; Mohades et al., 2015; Pliatsikas et al., 2015). But although at first pass these differences seem encouraging, it is problematic that in some cases the same metric under study (e.g., fractional anisotropy measuring directional diffusion of water in white matter) is higher in the bilingual group, and in other cases the metric is higher in the monolingual group. Sometimes these contradictory effects occur in the same fibre pathways. For example, Gold et al. (2013) show increased white matter in the IFOF for monolinguals while Luk, Bialystok et al. (2011) show a decrease. Thus, as in the morphologic and ERP literature, inconsistency overwhelms a clear and concise neurobiological story. Some methodological issues explaining such contradictions are likely to be unique to research on bilingual populations (e.g., the definition of bilingualism, or the age-group under study). Others may be specific to research using DWI (e.g., the post-processing pipeline, or the acquisition parameters of the scan), or to both (e.g., age is a methodological confound for both bilingual research and DWI). However, at present the research on structural differences offers an ambiguous and inconsistent story about the neurobiology of bilingualism more generally (García-Pentón et al., 2016), and does not strongly and reliably overlap with expectations from neurocognitive models of the bilingual advantage for executive function.

fMRI studies have been used to examine whether activation dynamics in brain regions associated with executive function during non-language tasks are different across bilingual and monolingual groups. In an often-cited and focused review, Pliatsikas and Luk (2016) examined a corpus of such studies and concluded, based on this evidence, that bilingual experience has consequences for brain activity in domain-general executive control regions beyond language processing. In some cases, the reviewed studies indeed report bilingual-monolingual differences in areas associated with executive control, including the right caudate nucleus (Mohades et al., 2014); anterior cingulate cortex (Garbin et al., 2010; Mohades et al., 2014); and left parietal lobule (Ansaldo et al., 2015, although no direct comparison between groups was provided; Luk et al., 2010). In addition, studies have identified differences in bilinguals and monolinguals in many other regions (e.g., in the bilateral cerebellum, bilateral superior temporal gyri, left supramarginal gyrus, bilateral postcentral gyri, and bilateral precuneus; Luk et al., 2010) that would not have been predicted by either the Adaptive Control model or the BAPPS model. It is not always clear, therefore, how differences between bilinguals and monolinguals reflect the functioning of brain networks involved in domain-general executive function. Furthermore, and similar to ERP studies, it is sometimes unclear whether an increase or decrease in these regions would reflect more efficient processing in bilinguals, and sometimes both directions have been observed (cf. Garbin et al., 2010 vs. Mohades et al., 2014).

#### fMRI and behavioural data

The interpretation of the direction of activation differences becomes even more difficult when the findings are either not accompanied by behavioural differences or accompanied by a behavioural disadvantage for the bilingual group. For example, Mohades et al. (2014) scanned two groups of bilingual children and a group of monolingual children while they performed a Simon task, and a Stroop task, in the MRI scanner. In prior behavioural studies, bilingual children show smaller switch costs and faster RTs on these tasks (Bialystok et al., 2004; Coderre & van Heuven, 2014). But in the study by Mohades and colleagues, the two bilingual groups actually showed significantly *worse* performance than the monolingual participants. Furthermore, the brain differences were not related directly to behavioural performance. This, coupled with the fact that the bilingual children actually performed worse on the tasks based on behavioural measurements, calls into question the degree to which this study provides neuroimaging support for a bilingual executive function advantage in Simon and Stroop tasks. This study is cited as evidence in favor of the BAPSS model because it shows that children *over-recruit* regions that are later engaged more efficiently by bilingual adults (Grundy et al., 2017). But given the performance difference, with bilinguals performing more poorly, it is difficult to fit this study within a model trying to explain bilingual *advantages* in executive function. This is a good illustration of the problem of an under-specified neurobiological theory, because essentially all findings fit the model.

Other studies find brain differences without behavioural differences (e.g., Ansaldo et al., 2015; Luk et al., 2010). Despite the absence of behavioural differences, the latter study by Luk et al. is cited to support the conclusion that “response inhibition and response selection are distinguishable but related processes; and the recruitment of the more distributed network for response selection by bilinguals suggests that they can rely on this network for interference suppression more successfully than monolinguals” (Pliatsikas & Luk, 2016, p. 700). The lack of a significant group difference might be due to the small sample size (*n* = 10), but regardless, the lack of a behavioural difference makes the neuroimaging data difficult to interpret. Unlike some studies reviewed above, the authors did examine the association between behavioural performance and brain activation. But again, there was no statistically significant relationship identified. Despite this, the authors reported that the pattern of brain-behaviour associations was different between the groups.

Similar problems plague other studies, even those conducted more recently. For example, a more recent study by DeLuca et al. (2020) using the flanker paradigm in an fMRI study of bilinguals also found no behavioural difference—that is, measures of bilingual language experience did not modulate behavioural performance on the task. In other studies using task-switching paradigms, group differences in response time were not statistically reliable, or direct comparison of activation differences was not statistically reliable across the groups, or both (Garbin et al., 2010; Rodríguez-Pujadas et al., 2013). An exception to this is the study by Gold et al. (2013), which did report a bilingual advantage for task switching, although this only occurred for an older bilingual group, but not for a younger bilingual group tested on the same task. Activation differences were found in left middle and inferior frontal gyrus, and anterior cingulate cortical regions predicted by the Adaptive Control model, but only for the older group. In terms of its overlap with the regions proposed by the Adaptive Control Model, this might provide the strongest combination of behavioural and brain differences, but it still provides only partial support for the predictions of the model because the finding only occurs in the older group. Thus, there is simultaneously evidence for and against the model predictions.

#### Reverse inference

The link between behaviour and brain is arguably critical for understanding the source of potential cognitive benefits of bilingualism, but research groups differ in terms of how they view the utility of behavioural data. Some have suggested that the lack of behavioural differences is an advantage, and argue that “equivalent performance in the two groups allows meaningful interpretation of the differences in functional neural correlates without the possible confound of behavioral differences” (Luk et al., 2010, p. 355). De Luca et al. (2020) suggest that neuroimaging data are more “granular” and by implication more sensitive and reliable, and Grundy et al. (2017) make a complementary argument, stating that, when investigating the bilingual advantage, matching behaviour allows brain differences to be discussed in the absence of a behavioural confound.

However, the opposite is arguably true. First, neuroimaging data tend to be *noisier* and *less* sensitive than behavioural data (e.g., ADHD-200 consortium, Brown et al., 2012). Second, and more importantly, we argue that the behavioural difference is not a confound, but rather it is a condition on which the study is predicated. A coherent model of how bilingual experience shapes the specialization of neural regions involved in domain general executive function should at a minimum be able to relate performance differences to regional activity proposed to be involved in the executive function processes of interest. In fact, any model claiming a behavioural advantage for executive function in bilinguals should be able to show evidence of such an advantage in the sample under investigation. As an analogy, a pharmaceutical company would not be providing convincing evidence of a new performance enhancing drug if they failed to show that it enhances performance, even if they could reliably show differences in activation patterns in brain circuitry between treated and non-treated participants.

In summary, although the conclusion of some research groups (e.g., DeLuca et al., 2020; Grundy et al., 2017; Pliatsikas & Luk, 2016) is that bilingual experience has consequences for neural processing during executive control tasks, the evidence is inconsistent, not always supported by behavioural differences or correlations with behavioural patterns, and suffers from critical threats to internal validity. One of the biggest threats to internal validity, which applies to both the fMRI and the structural imaging findings, is the strong reliance on what Poldrack (2006) called “reverse inference.” Put simply, this is the process of reasoning backwards from the presence of some brain activation or structural difference to the engagement of or difference in a particular cognitive function.

Why is reverse inference reasoning a threat to internal validity? At a definitional level, internal validity is about causal inferences, or more specifically that any observed covariation between A and B reflects a causal relationship (Shadish et al., 2002). Because it is a characteristic of a knowledge claim (Shadish, 1995), it is not inherent to research design, but the degree to which a claim has high internal validity is tightly bound to research design. Thus, to support valid inference of causality, the research design must maximize three tenets of internal validity: (1) A must precede B in time; (2) A must covary with B (which is implied), and (3) no other explanations for the relationship are plausible.

When reverse inference reasoning is employed, it is often done without regard to design, and in most cases, it violates at least two of the tenets described above, and sometimes all three. Further, as Poldrack (2006) notes, fundamentally, reverse inference is a deductively invalid line of argument; that is, it is akin to the logical fallacy of affirmation of the consequent.(Affirmation of the consequent is a formal logical fallacy. It takes the form “IF P, THEN Q. Q, THEREFORE P.” For example, “IF an animal is a cat, THEN it has a tail. My dog has a tail, THEREFORE it is a cat.”) It begins with the assumption of a one-to-one mapping between brain activity / activity differences and a specific behaviour, taking the argument form, “If cognitive process A is engaged, then brain area B is active.” Then it applies the logical fallacy, “If brain area B is active, cognitive process A is engaged” (i.e., affirmation of the consequent). If the association between A and B were exclusive, this would not necessarily be invalid. That is, when B occurs IF AND ONLY IF A occurs, there is the establishment of temporal precedence and covariance, and a mitigation of alternative explanations for the association. But for brain imaging data, this is rarely, if ever, the case. Even in primary cortical areas, exclusivity is not established (e.g., activity in primary visual cortex does not imply the person is seeing anything physically in the environment because visual cortex can show activity during visual hallucinations in the absence of sensory input; Pajani et al., 2015). In regions associated with higher-order cognitive processes, selectivity of activation associated with a particular process is very difficult to establish (Poldrack, 2006).

Despite this shortcoming, in the set of neuroimaging studies we reviewed, the logic of reverse inference was predominant. Thus, when behavioural differences are not established or even tested, or the results are in the opposite direction from what was expected (i.e., bilinguals perform worse), or when there is no tested or established association between brain and behaviour, there cannot be a valid statement about how fMRI or structural MRI data relate to cognitive benefits of bilingualism. At best, using this line of reasoning is useful as a statement of probability (Poldrack, 2006), but noting activation or structural differences in a region cannot, by itself and without a link to behaviour, provide strong evidence that a cognitive process is at work.

#### Individual differences in language experiences

Assessing whether there are structural or functional brain differences between bilinguals and monolinguals is an interesting question on its own. Indeed, there is a large amount of literature focusing on differences between bilinguals and monolinguals without making claims regarding behavioural consequences or bilingual advantages. Recent frameworks have been proposed to describe and understand how bilingualism might change brain structure and functions (e.g., Pliatsikas, 2019), including a focus on different types of language learners and bilinguals. Empirical work has started to examine how different bilingual experiences can shape brain structure and function. For example, DeLuca et al. (2019) studied how L2 age of acquisition, L2 length of immersion, L2 use at home, and L2 use in social settings related to brain structure and connectivity. Their results showed relationships between the various language experiences examined and structure and connectivity measures, highlighting the importance of studying individual differences in future research. Other recent studies have also started to focus on individual differences between bilinguals in fMRI studies on executive control (e.g., Claussenius-Kalman et al., 2020; Del Maschio et al., 2020; DeLuca et al., 2020; Gullifer et al., 2018). These studies will be of great use to further develop theories on neural changes in response to language learning and/or bilingualism (e.g., Pliatsikas, 2019). In line with similar developments in behavioural studies, these are very important and promising pathways. However, similar to behavioural research, we do not know enough about how individual differences modulate *language* control in the brain to formulate concise hypotheses about how these individual differences might modulate the neural correlates of executive control. Both behavioural and neuroimaging research on individual differences in *language* control is pivotal if we want to understand how bilingualism might modulate the structure and function of the brain in relation to non-linguistic control.

## MOVING FORWARD

There is a clear and undisputable advantage that comes with bilingualism: being bilingual and being able to communicate and connect with more people. Bilingualism and bilingual education should therefore be promoted, regardless of the putative cognitive consequences. In this review we focused on key issues affecting behavioural and neuroimaging research on bilingualism and executive control: the need for falsifiable theories and the need for a better understanding of bilingual language control. The development of vague theoretical frameworks and loose interpretation of behavioural data mean that existing theories are becoming more difficult to falsify. We are not arguing that changes in theoretical frameworks or hypotheses are problematic. To advance as a field, new data should stimulate the formulation of new hypotheses and theories need to be updated based on new findings. The increasing emphasis on individual bilingual experiences, moving away from comparisons between bilinguals and monolinguals as groups without clear definitions of their language profile, is also laudable. However, the research into bilingual *language* control and the relationship with individual bilingual experiences is still in its infancy. Understanding this relationship is crucial for the argument that language experiences might be related to executive control. Before we can form clear hypotheses, we first need to clarify the link between *language* control and *language* experiences. Until we know more about bilingualism, bilingual language control, and structural and functional networks related to bilingualism, theories and hypotheses will remain vague. This means theories can be supported regardless of their results and cannot be falsified. For both behavioural and neuroimaging research on this topic to develop and improve, more precise theories on bilingual experiences and language control (as well as the relationship with cognitive control) are needed.

To move forward, especially with a focus on individual differences between bilinguals, we need more multi-lab collaborations in which researchers a priori agree on (and pre-register) the type of participants, measures of interest, data collection, data analysis, and data interpretation. Such a priori agreement will help with the development of clear, testable hypotheses and can help to overcome the multiple interpretations that are possible when, for example, different tasks show different findings. Such a collaboration across research groups will allow for a more unbiased interpretation of data, which in turn can help to formulate clearer theories. Moreover, multi-lab collaborations are especially important if we want to compare different types of bilingualism. To directly compare different bilinguals and the role of individual experiences, access is needed to multiple bilingual populations who complete the exact same study and are analysed in exactly the same way.

It is not a solution to just keep adding data. For the research field to progress, we need to take a step back. We need to study the cognitive and neural mechanisms underlying language control. This knowledge can then be used to formulate clearer theories and hypotheses, which in turn need to be tested in collaborations across research groups.

## ACKNOWLEDGMENTS

The first author received funding from the European Union’s Horizon 2020 research and innovation programme under the Marie Skłodowska-Curie grant agreement number 743691. The last author received funding from the Basque Government (2018–2021 BERC), the Agencia Estatal de Investigacion: The Severo Ochoa Programme for Centres/Units of Excellence (SEV-2015-490) and grant (RTI2018-093547-B-I00).

## FUNDING INFORMATION

Angela de Bruin, H2020 Marie Skłodowska-Curie Actions (https://dx.doi.org/10.13039/100010665), Award ID: 743691. Manuel Carreiras, Eusko Jaurlaritza (https://dx.doi.org/10.13039/501100003086), Award ID: 2018-2021 BERC. Manuel Carreiras, Agencia Estatal de Investigación (https://dx.doi.org/10.13039/501100011033), Award ID: SEV-2015-490. Manuel Carreiras, Agencia Estatal de Investigación (https://dx.doi.org/10.13039/501100011033), Award ID: RTI2018-093547-B-I00.

## AUTHOR CONTRIBUTIONS

**Angela de Bruin**: Conceptualization: Equal; Writing – original draft: Equal; Writing – review & editing: Equal. **Anthony Dick**: Conceptualization: Equal; Writing – original draft: Equal; Writing – review & editing: Equal. **Manuel Carreiras**: Conceptualization: Equal; Writing – original draft: Equal; Writing – review & editing: Equal.

## TECHNICAL TERMS

Bilingual advantage: Better performance (reaction times or/and accuracy) of bilinguals as compared to monolinguals in cognitive tasks.
EEG: Electroencephalography is a technique to record electrical activity of the brain with the electrodes placed along the scalp.
MRI: Magnetic Resonance Imaging is an imaging technique used in radiology to form pictures of the anatomy and the physiological processes of the body.
fMRI: Functional Magnetic Resonance Imaging measures brain activity by detecting changes associated with blood flow.
DWI: Diffusion-weighted Imaging is the use of specific MRI software that generates images from the diffusion of water molecules.
Brain plasticity: The ability of neural networks in the brain to change and reorganize, such as when learning a new ability like a second language.
